# Bridging preclinical and clinical gut microbiota research using the *ex vivo* SIFR^®^ technology

**DOI:** 10.3389/fmicb.2023.1131662

**Published:** 2023-04-14

**Authors:** Pieter Van den Abbeele, Stef Deyaert, Clémentine Thabuis, Caroline Perreau, Danica Bajic, Eva Wintergerst, Marie Joossens, Jenni Firrman, Dana Walsh, Aurélien Baudot

**Affiliations:** ^1^Cryptobiotix SA, Ghent, Belgium; ^2^Nutrition and Health R&D, Roquette, Lestrem, France; ^3^Glycom A/S-DSM Nutritional Products Ltd., Hørsholm, Denmark; ^4^DSM Nutritional Products, Kaiseraugst, Switzerland; ^5^Laboratory of Microbiology, Department of Biochemistry and Microbiology, Ghent University, Ghent, Belgium; ^6^United States Department of Agriculture, Agricultural Research Service, Eastern Regional Research Center, Wyndmoor, PA, United States; ^7^CosmosID, Germantown, MD, United States

**Keywords:** microbiota, gut, prebiotic, SIFR^®^, *ex vivo*

## Abstract

**Introduction:**

While modulation of the human adult gut microbiota is a trending strategy to improve health, the underlying mechanisms are poorly understood.

**Methods:**

This study aimed to assess the predictive value of the *ex vivo*, reactor-based, high-throughput SIFR^®^ (Systemic Intestinal Fermentation Research) technology for clinical findings using three structurally different prebiotics [inulin (IN), resistant dextrin (RD) and 2′-fucosyllactose (2′FL)].

**Results:**

The key finding was that data obtained within 1–2 days were predictive for clinical findings upon repeated prebiotic intake over weeks: among hundreds of microbes, IN stimulated *Bifidobacteriaceae*, RD boosted *Parabacteroides distasonis*, while 2′FL specifically increased *Bifidobacterium adolescentis* and *Anaerobutyricum hallii*. In line with metabolic capabilities of these taxa, specific SCFA (short-chain fatty acids) were produced thus providing insights that cannot be obtained *in vivo* where such metabolites are rapidly absorbed. Further, in contrast to using single or pooled fecal microbiota (approaches used to circumvent low throughput of conventional models), working with 6 individual fecal microbiota enabled correlations that support mechanistic insights. Moreover, quantitative sequencing removed the noise caused by markedly increased cell densities upon prebiotic treatment, thus allowing to even rectify conclusions of previous clinical trials related to the tentative selectivity by which prebiotics modulate the gut microbiota. Counterintuitively, not the high but rather the low selectivity of IN caused only a limited number of taxa to be significantly affected. Finally, while a mucosal microbiota (enriched with *Lachnospiraceae*) can be integrated, other technical aspects of the SIFR^®^ technology are a high technical reproducibility, and most importantly, a sustained similarity between the *ex vivo* and original *in vivo* microbiota.

**Discussion:**

By accurately predicting *in vivo* results within days, the SIFR^®^ technology can help bridge the so-called “Valley of Death” between preclinical and clinical research. Facilitating development of test products with better understanding of their mode of action could dramatically increase success rate of microbiome modulating clinical trials.

**Graphical Abstract:**
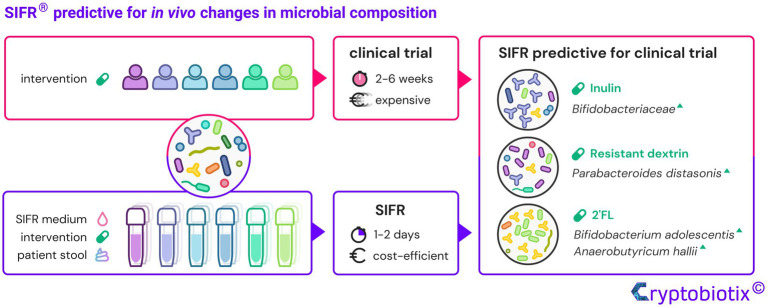

## Introduction

Over the past decades, the importance of the gut microbiota for human health has become increasingly apparent in the context of intestinal diseases (Lloyd-Price et al., 2019; Rebersek, 2021) but, among others, also brain-related diseases (Shen et al., 2021). A key function of the gut microbiota is to ferment plant- and host-derived glycans into short-chain fatty acids (SCFA)(Flint et al., 2012) such as acetate, propionate, and butyrate that each contribute to particular health benefits (Rivière et al., 2016). As a result, glycans received major attention as modulators of the gut microbiota and consequently human health. Some glycans classify as prebiotics, i.e., substrates that are selectively utilized by host microorganisms conferring a health benefit (Gibson et al., 2017). While fructans such as inulin (IN) are among the traditional prebiotics, novel classes are human milk oligosaccharides (HMOs) [such as 2′fucosyl-lactose (2′FL)] (Gibson et al., 2017) and resistant dextrin (RD)(Lefranc-Millot et al., 2012; Włodarczyk and Śliżewska, 2021).

By systematically sampling various sites of the human gut, Lavelle et al. (2015) recently demonstrated that interpersonal differences are the major source for variation of gut microbiota composition *in vivo*, followed by differences between luminal and mucosa-associated microbiota. These interpersonal differences impact the outcome of interventions and are thus highly relevant to the efficacy of prebiotic treatments (Healey et al., 2017). A recent study by Nguyen et al., (2020) highlighted that metabolic functions relevant for physiological effects of arabinoxylans were indeed donor-dependent, stressing that interpersonal differences could be a key contributor for the inconsistent reports on benefits of fiber intake (Armet et al., 2020).

Clinical trials are essential to demonstrate health benefits, however, given the large intrinsic variability between humans and the inaccessibility of the site of fermentation where metabolites are rapidly absorbed, they are less suited for elucidating the impact of prebiotics on microbial activity and composition (Ruppin et al., 1980; Delcour et al., 2016). Only exceptional study methods, such as the use of stable-isotope dilution, allow estimation of *in vivo* SCFA production upon IN intake (Boets et al., 2015). This method is limited by the uncertainty of SCFA bioavailability that is estimated based on literature data and assumed identical for all test subjects. Alternatively, intake of high doses of prebiotics (e.g., 35 g/day) allows identification of changes in fecal SCFA levels (Deehan et al., 2020). However, while not allowing estimation of *in vivo* SCFA production, such high doses are typically not economically viable in a final, commercial product, and can also lead to tolerance issues (Bonnema et al., 2010).

Preclinical studies have the potential to complement clinical trials. However, as reviewed by Seyhan et al., there is increasing awareness of the so-called “Valley of Death” between preclinical and clinical research (Seyhan, 2019), characterized by poor translation of laboratory findings to human outcomes. In gut microbiome research, the first limitation with the current *in vitro/ex vivo* models is the drastic compositional alteration of the *in vivo*-derived microbial community toward an *in vitro*-adapted one. This is highly pronounced for short-term models where, within a timeframe as short as 24 h, fast-growing, aerotolerant taxa dominate the communities that contain, e.g., 50% *Enterobacteriaceae* (Van den Abbeele et al., 2020a), 75–80% Proteobacteria (Biagini et al., 2020), 75% *Veillonellaceae* (Gaisawat et al., 2019), or 60–70% *Escherichia-Enterococcus-Streptococcus* (O’Donnell et al., 2018). Similarly, the current generation of long-term gut models, aiming to simulate an average human individual, impose defined nutritional and environmental conditions, thus enriching taxa that thrive under these specific conditions (Rajilić-Stojanović et al., 2010; Van den Abbeele et al., 2010), as quickly as within 3 days (Van den Abbeele et al., 2013). A second limitation is the common low-throughput of current gut microbiome models, resulting in smaller-scale, less robust experimental designs, lacking parallel controls and/or technical/biological replicates. To bypass the representation of interpersonal differences, samples of multiple subjects are sometimes pooled (Aguirre et al., 2014). However, there is hardly any available data on the impact of pooling on research outcomes and such an approach is controversial, as it erases the interpersonal differences and promotes niche competition.

As improved *ex vivo* studies are required to optimize *in vivo* intestinal microbiome modulation, the aim of the current study was to validate a miniaturized, bioreactor-based, high-throughput, *ex vivo* gut microbiome platform, i.e., the SIFR^®^ (Systemic Intestinal Fermentation Research; pronounced “*cipher*,” IPA: [ˈ*sī-f*′*r*]) technology. The *in vivo* predictivity of the SIFR^®^ technology was extensively and comprehensively examined using three well-known prebiotics (IN, RD, and 2′FL) that differ structurally at a fundamental level, and bench-marked against available high-quality clinical data (Elison et al., 2016; Vandeputte et al., 2017a; Iribarren et al., 2020; Thirion et al., 2022). Technical aspects were further assessed including technical reproducibility as well as the impact of different donor scenarios (replicates from one donor, different donors, replicates from pooled sample) and the simulation of mucosa-associated microbiota.

## Materials and methods

### Test products

Test products evaluated were: IN from chicory (I2255, Merck, Overijse, Belgium), RD NUTRIOSE^®^ FB06, Roquette, Lestrem, France) and 2′FL (GlyCare 2FL 9000, Glycom A/S-DSM Nutritional Products Ltd., Hørsholm, Denmark). A no substrate control (NSC), in which the microbial inoculum was grown in absence of additional test products, yet in presence of an optimized nutritional medium was used as control.

### *In vivo-*derived microbiota

Based on the data of Lavelle et al. demonstrating minor longitudinal differences along the colon of healthy individuals (Lavelle et al., 2015), fecal samples were used as a *proxy* for the colonic microbiota. The 18 fecal donations scored between 3 and 4 on the Bristol stool score (BSS) scale suggesting they were not subject to long distal transit times (Falony et al., 2016; Müller et al., 2019). The donors complied to the following criteria: age 25–65, no antibiotic/probiotic use in 3 months prior to study, no gastrointestinal complaints nor diagnosed disorders (cancer, ulcers, IBD), non-smoking, alcohol consumption <3 units/day and BMI <30 and >18.5. All 18 samples consistently contained taxa belonging to key phyla, i.e., *Actinobacteria (Actinomycetota)*, *Bacteroidetes (Bacteroidota)*, *Firmicutes (Bacillota)* and *Verrucomicrobia* (Supplementary Figure S1).

### Experimental design

**Figure 1:**
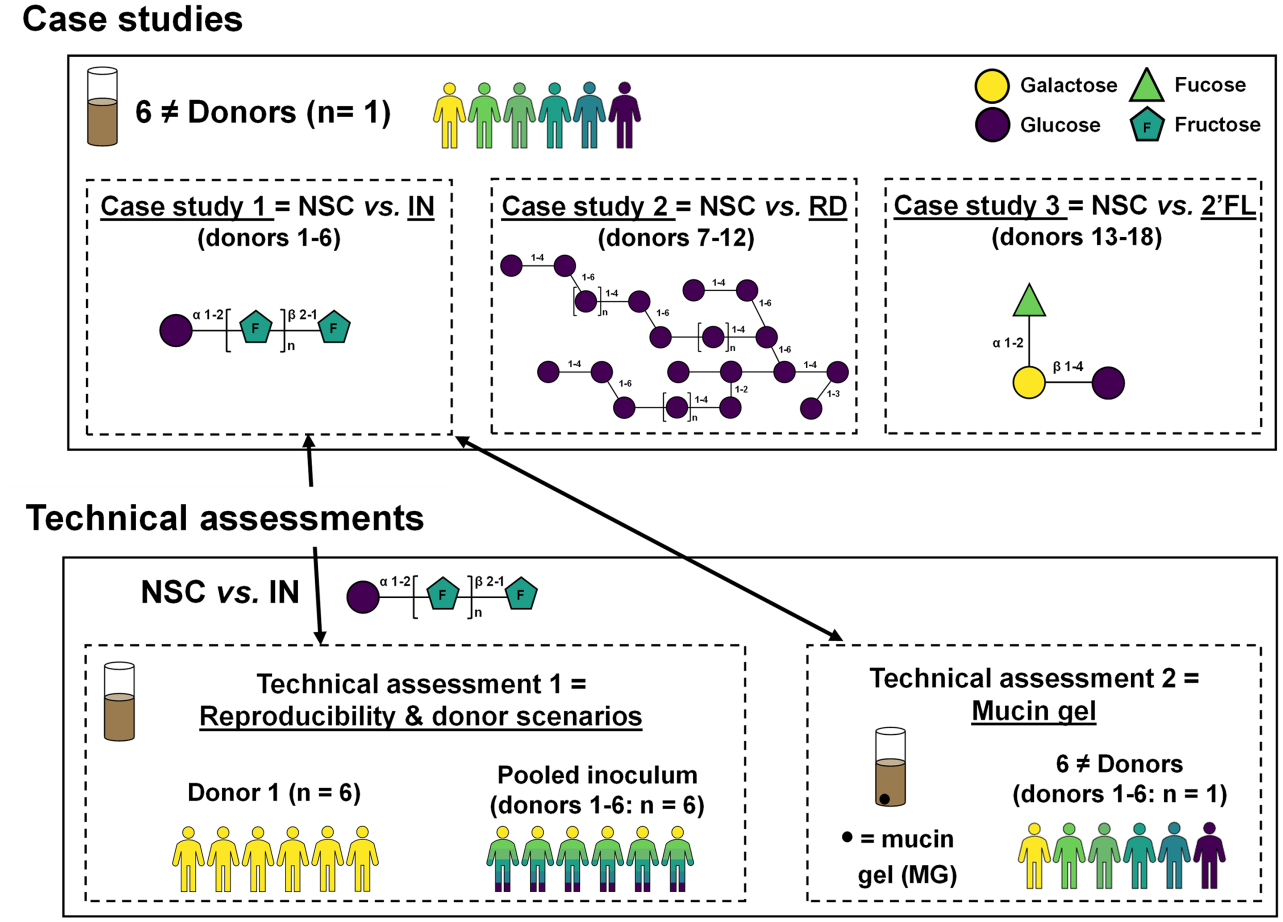
Study design of three case studies with IN, RD and 2′FL to assess model predictivity and two technical assessments focussing on model reproducibility/donor scenarios and simulation of a mucin gel.

Three case studies were performed to assess predictivity of the SIFR^®^ technology, followed by two additional technical assessments (Figure 1). During each case study, 6 different donors (*n* = 1) were used to assess the impact of IN (case study 1), RD (case study 2) and 2′FL (case study 3). Technical assessment 1 involved testing two more donor scenarios (one donor and a pooled sample) in 6 technical replicates, thus not only allowing to evaluate technical reproducibility, but also, along with data of case study 1 (performed with 6 donations used to generate the pooled sample), allowing to extensively evaluate different donor scenarios. Finally, technical assessment 2 involved the simulation of a mucin gel to assess how IN modulates not only luminal, but also mucosal microbiota in SIFR^®^ reactors.

### SIFR^®^ technology

Individual bioreactors were processed in parallel in a bioreactor management device (Cryptobiotix, Ghent, Belgium). Each bioreactor contained 5 ml of nutritional medium-fecal inoculum blend supplemented with 5 g prebiotic/L, then sealed individually, before being rendered anaerobic. Blend M0003 was used for the case studies and technical assessment 1 and blend M0012 for technical assessment 2 (Cryptobiotix, Ghent, Belgium). During technical assessment 2, a mucin gel was prepared by boiling a solution of 5% mucin type II (M2378; Merck, Overijse, Belgium) and 1% agar for 2 min. Upon cooling, the pH was adjusted to 6.8 ± 0.1. Mucin droplets were introduced in polyethylene carriers with volume of 0.07 cm^3^. After preparation, bioreactors were incubated under continuous agitation (140 rpm) at 37°C for 24 h, except for case study 2, where a more integrative time point of 48 h was implemented (MaxQ 6,000, Thermo Scientific, Thermo Fisher Scientific, Merelbeke, Belgium). Upon gas pressure measurement in the headspace, liquid samples were collected for subsequent analysis.

### Fundamental fermentation parameters

SCFA (acetate, propionate, butyrate and valerate) and branched-chain fatty acids (bCFA; sum of isobutyrate, isocaproate and isovalerate) were determined *via* GC with flame ionization detection (Trace 1300, Thermo Fisher Scientific, Merelbeke, Belgium), upon diethyl ether extraction as previously described (De Weirdt et al., 2010). pH was measured using an electrode (Hannah Instruments Edge HI2002, Temse, Belgium).

### Microbiota phylogenetic analysis: Quantitative shallow shotgun sequencing

Quantitative data was obtained by correcting abundances [%; shallow shotgun sequencing (3 M reads)] with total cell counts for each sample (cells/mL; flow cytometry), resulting in estimated cell counts/mL. Mucosal samples were not analyzed with flow cytometry as these cannot be converted to single cell suspensions. Data of mucosal microbes was expressed as relative abundances (%).

DNA was extracted *via* the SPINeasy DNA Kit for Soil (MP Biomedicals, Eschwege, Germany), according to manufacturer’s instructions. Subsequently, DNA libraries were prepared using the Nextera XT DNA Library Preparation Kit (Illumina, San Diego, CA, United States) and IDT Unique Dual Indexes with total DNA input of 1 ng. Genomic DNA was fragmented using a proportional amount of Illumina Nextera XT fragmentation enzyme. Unique dual indexes were added to each sample followed by 12 cycles of PCR to construct libraries. DNA libraries were purified using AMpure magnetic Beads (Beckman Coulter, Brea, CA, United States), eluted in QIAGEN EB buffer, quantified using a Qubit 4 fluorometer and a Qubit dsDNA HS Assay Kit, and sequenced on an Illumina Nextseq 2000 platform 2 × 150 bp. Unassembled sequencing reads were converted to relative abundances (%) using the CosmosID-HUB Microbiome Platform (CosmosID Inc., Germantown, MD, United States; Hasan et al., 2014; Agarwal et al., 2022). For total cell count analysis, liquid samples were diluted in anaerobic phosphate-buffered saline (PBS), after which cells were stained with SYTO 16 at a final concentration of 1 μM and counted *via* a BD FACS Verse flow cytometer (BD, Erembodegem, Belgium). Data was analyzed using FlowJo, version 10.8.1.

### Statistical analysis

All univariate and multivariate analyses were performed by GraphPad Prism (v9.3.1; www.graphpad.com), while Regularized Canonical Correlation Analysis (rCCA) was executed using the mixOmics package with the shrinkage method for estimation of penalization parameters (version 6.16.3) in R (4.1.1; www.r-project.org) (Rohart et al., 2017). Treatment effects were compared with the NSC using ANOVA analysis and *p*-values were corrected with Benjamini and Hochberg (1995) (corrected FDR = 0.05 for technical replicates and 0.10 for biological replicates). Paired testing (repeated-measures ANOVA) was performed for setups considering 6 donors in *n* = 1. For analysis of microbial composition, three measures were taken. First, aforementioned statistical analysis was performed on the log_10_-transformed values. Second, a value of a given taxonomic group below the limit of detection (LOD) was considered equal to the overall LOD according to the procedure elaborated in Supplementary Figure S2. Finally, a threshold was set in order to retain the 100 most abundant species in the analysis, to avoid excessive *p*-values corrections.

## Results and discussion

### Case studies

#### Sustained similarity between original donor microbiota and SIFR^®^ technology, an accurate *ex vivo* gut microbiome-testing platform (case study 1)

**Figure 2:**
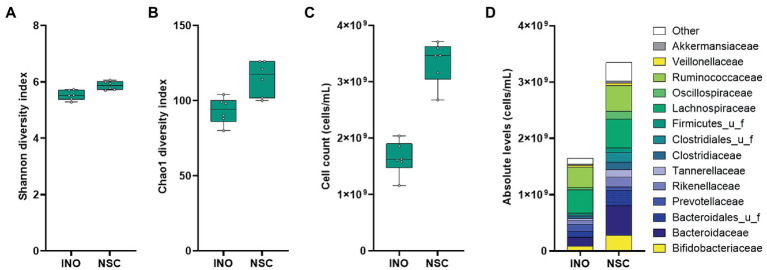
Case study 1: Microbial diversity **(A,B)**, cell counts (cells/mL) **(C)** and average microbial composition at family level (cells/mL) **(D)** of the *in vivo*-derived inocula (INO) and upon 24 h of incubation in the SIFR^®^ technology in absence of prebiotic treatment (NSC) (*n* = 6).

A comparison between the original sample and the same sample incubated for 24 h using the SIFR^®^ technology was performed first, to assess the accuracy of the technology for mimicking *in vivo* conditions. A diverse range of *in vivo*-derived gut microbes endured over the 24 h incubation period, maintaining microbial diversity, both in terms of species richness and evenness (Figures 2A,B). Under control conditions (NSC), the total cell numbers even increased from 0 h (INO) to 24 h with a factor of 2.06 (±0.25) (Figure 2C). Importantly, at the end of the 24 h NSC incubation, the microbial composition reflected to the original inoculum (INO; Figure 2D). This accurate preservation of *in vivo*-derived microbiota for the entire duration of the experiment classifies the application of SIFR^®^ technology as an *ex vivo* study, which is a study that uses an artificial environment outside the human body with minimum alteration of natural conditions. Such sustained similarity is fundamentally different from consistent biases observed for the current generation of *in vitro* gut models (Rajilić-Stojanović et al., 2010; Van den Abbeele et al., 2010, 2013, 2020a; O’Donnell et al., 2018; Gaisawat et al., 2019; Biagini et al., 2020).

#### SIFR^®^ technology provided data predictive of clinical outcome of IN on gut microbiota composition and generated insights into metabolites intractable *in vivo* (case study 1)

**Figure 3:**
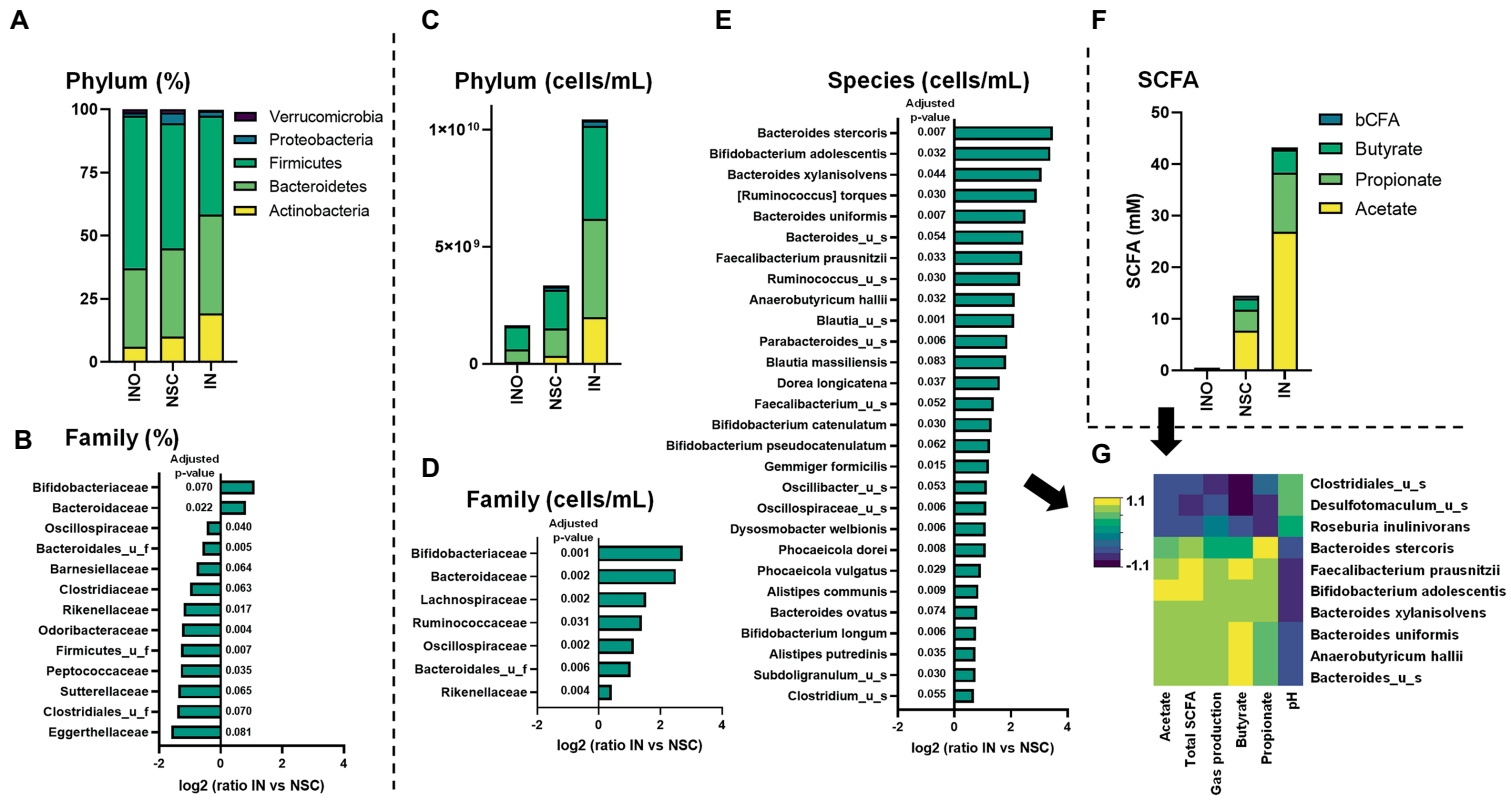
Case study 1: Average levels (*n* = 6) of microbial phylum [proportional **(A)**/absolute **(C)**] and SCFA **(F)** for the *in vivo*-derived inocula (INO) and upon 24 h of incubation in the SIFR^®^ technology (NSC or IN treatment). The families that were significantly affected by IN treatment, either when statistics were based on proportional and absolute data are shown in, respectively, **(B,D)** (expressed as log_2_(ratio of abundance upon IN treatment compared to NSC), along with the corresponding adjusted *p*-value). Further, IN also significantly impacted the absolute levels of several species **(E)**, resulting in marked correlations between specific species and metabolic markers (threshold >0.8) **(G)**.

IN primarily increased relative abundances of Actinobacteria, due to the stimulation of *Bifidobacteriaceae* (Figures 3A,B). This is in line with findings of Vandeputte et al. (2017a) which demonstrated that the key impact of inulin intervention using next-generation sequencing (%) is an increase in *Bifidobacteriaceae*. While such *in vivo* observations were obtained upon 4 weeks of repeated daily administration (12 g/day), the SIFR^®^ study only lasted for 24 h, displaying the efficiency at which data predictive for *in vivo* outcomes can be obtained using an *ex vivo* technology.

As reviewed by Delcour et al., a key limitation of *in vivo* studies is the inability to analyze the most prominent functional changes induced by prebiotics, i.e., increased production of SCFAs, given their rapid absorption/consumption *in vivo* (Delcour et al., 2016). Using the SIFR^®^ technology, it was demonstrated that IN significantly increased SCFA production for each of the 6 donors tested (Figure 3F; Supplementary Figure S3). In line with *in vivo* findings (Boets et al., 2015), IN most strongly increased acetate. Further, also propionate and butyrate production was enhanced. Further, marked interpersonal differences were observed in terms of how IN stimulated propionate (Supplementary Figure S3D) and butyrate (Supplementary Figure S3E), suggesting a low selectivity of IN. By performing *ex vivo* studies in parallel to clinical studies, unique insights in the production of metabolites can be generated that could explain *in vivo* observations such as responders/non-responders caused by interpersonal differences.

#### Combining SIFR^®^ technology with quantitative sequencing enabled insights into mechanism of action of IN-gut microbiome interplay (case study 1)

While quantitative sequencing has been introduced for the analysis of *in vivo* samples as a means to further discriminate enterotypes (Vandeputte et al., 2017b), it is particularly useful in *ex vivo* studies where, unlike *in vivo*, simulated colonic volumes are exactly known, allowing to accurately account for changes in microbial loads (Van den Abbeele et al., 2020b). Here, quantitative sequencing removed the large bias caused by the drastically increased cell counts upon IN treatment, as visualized at the phylum level (Figure 3C). This revealed that, in contrast to aforementioned conclusions based on relative data that exclusively identified a bifidogenic effect, a broad spectrum of families benefited from IN administration (Figure 3D), including families that, based on relative data, were supposedly unaffected (e.g., *Lachnospiraceae, Ruminococcaceae*) or even inhibited by IN (e.g., *Oscillospiraceae* and *Rikenellaceae*). Likewise, 28 species significantly increased upon IN treatment (Figure 3E), including among others *Bifidobacterium adolescentis* that was responsible for the marked bifidogenic effect of IN.

Further, correlations were established that support the mode of action. Butyrate production upon IN treatment correlated with *Faecalibacterium prausnitzii* and *Anaerobutyricum hallii* [formerly known as *Eubacterium hallii* (Shetty et al., 2018)], species known to produce butyrate *via* the butyryl-CoA:acetate CoA-transferase route (Louis and Flint, 2017). Further, propionate production correlated with *Bacteroides stercoris*, a species known to produce propionate *via* the succinate pathway (Johnson et al., 1986; Louis and Flint, 2017).

By accounting for interpersonal differences, the SIFR^®^ technology not only generated predictive findings, but also allowed for the formulation of a hypothesis regarding the mode of action. Understanding the mechanism by which microbiome-targeted products exert beneficial effects is essential to increase success rates of clinical studies and provide understanding when such studies failed (e.g., when a specific species is absent, it could explain non-response).

#### SIFR^®^ technology predicted compositional shifts in the gut microbiota induced by RD and 2′FL (case studies 2 and 3)

**Figure 4:**
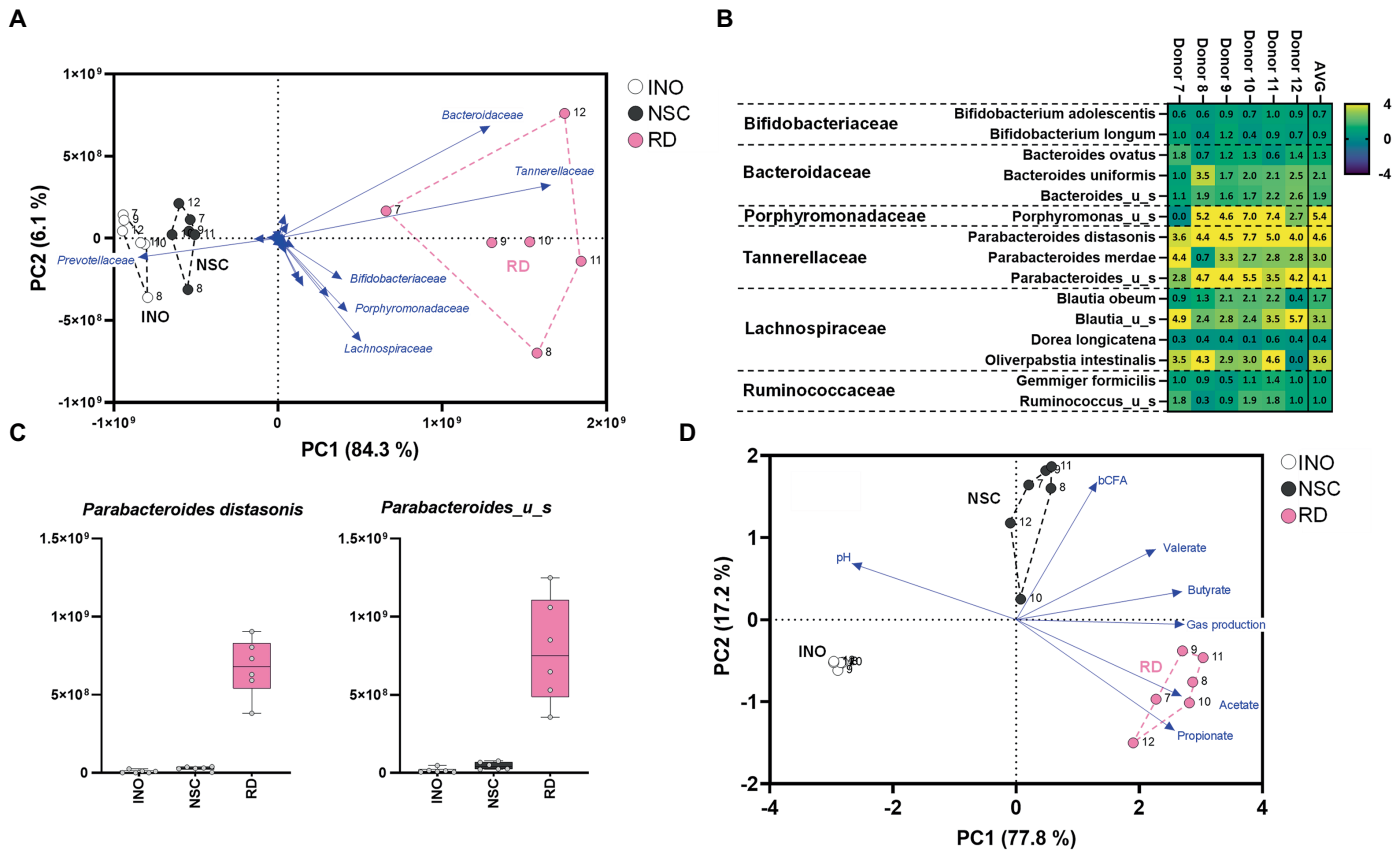
Case study 2: PCA based on microbial composition at family level (cells/mL) **(A)** and fundamental fermentation parameters **(D)** for the *in vivo*-derived inocula (INO) and upon 48 h of incubation in the SIFR^®^ technology (NSC and RD treatment). Multiple species were significantly affected upon RD treatment with some inter-donor differences **(B)**, additionally highlighted for the most strongly affected *Tannerellaceae* species **(C)**.

Resistant dextrin (RD) is a soluble fiber that is well tolerated and exerts health benefits by increasing satiety and improving both insulin resistance and determinants of metabolic syndrome (Li et al., 2010; Guérin-Deremaux et al., 2011). RD highly and specifically stimulates *Parabacterodes distasonis* from 0.92% at baseline up to 7.2% of the gut microbiota upon repeated intake by human adults over 6 weeks [20 g/day during final 4 weeks; (Thirion et al., 2022)]. A single intake study using the SIFR^®^ technology (48 h) demonstrated that RD treatment markedly increased *Lachnospiraceae* and especially *Tannerellaceae* levels (Figure 4A). At species level, *Parabacteroides distasonis* (Figures 4B,C; Supplementary Figure S4A) increased from 0.97% in the NSC up to 9.3% for RD treatment, thus highly accurately mirroring aforementioned clinical data (Thirion et al., 2022). In line with metabolic capabilities of *Parabacteroides distasonis* to produce acetate and propionate (Gotoh et al., 2017), RD strongly increased these SCFA (Figure 4D; Supplementary Figure S5). The rCCA confirmed this link and additionally revealed a correlation between (i) *Bacteroides uniformis* and propionate, and (ii) *Porphyromonas*_u_s and butyrate. *Bacteroides uniformis* has indeed been identified as potent dextrin fermenting species (Reichardt et al., 2018), able to produce propionate *via* the succinate pathway (Louis and Flint, 2017). Further, the unclassified *Porphyromonas* species belongs to the *Porphyromonadacae* family which contains species able to produce butyrate *via* the acetyl-CoA route (Vital et al., 2017), thus further supporting the involvement of particular members of this family in butyrate production from RD.

**Figure 5:**
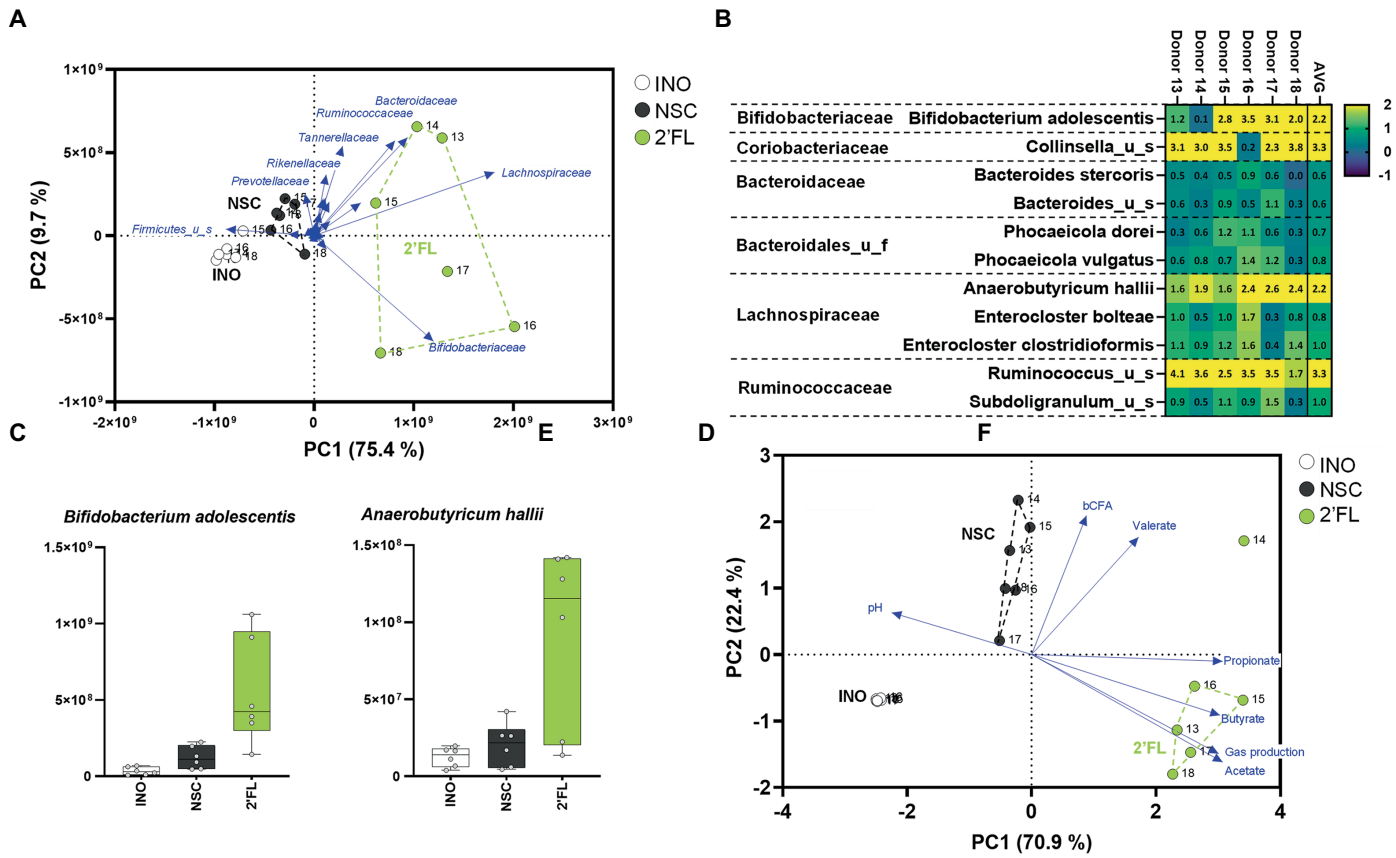
Case study 3: PCA based on microbial composition at family level (cells/mL) **(A)** and fundamental fermentation parameters **(D)** for the *in vivo*-derived inocula (INO) and upon 24 h of incubation in the SIFR^®^ technology (NSC and 2′FL treatment). Multiple species were significantly affected upon 2′FL treatment with some inter-donor differences **(C)**, additionally highlighted for the most strongly affected *Bifidobacteriaceae* and *Lachnospiraceae* species **(D)**.

A third case study was performed with 2′FL, an abundant HMO that exerts potent bifidogenic effects (related to stimulation of *Bifidobacterium adolescentis*) when dosed to human adults over 2 weeks [at 5, 10, or 20 g/day; (Elison et al., 2016)]. Furthermore, when administered to IBS patients (over 4 weeks at 5 or 10 g/day), bifidogenic effects were accompanied by an increase of *Anaerobutyricum hallii* (Iribarren et al., 2020). A single intake study using the SIFR^®^ technology (24 h) revealed that 2′FL markedly increased levels of *Bifidobacteriaceae* and *Lachnospiraceae* (Figure 5A) due to increases of *B. adolescentis* and *A. hallii* (Figures 5B,C). In line with the metabolic capabilities of *B. adolescentis* and *A. hallii* to, respectively, produce acetate (De Vuyst et al., 2014) and propionate/butyrate (Engels et al., 2016), 2′FL strongly increased the production of these SCFA (Figure 5D; Supplementary Figure S7). Two mechanisms have been identified that highlight the metabolic complementarity of *B. adolescentis* and *A. hallii*. First, *Bifidobacterium* species produce acetate and lactate (De Vuyst et al., 2014) that can be consumed by *A. hallii* to produce butyrate (Engels et al., 2016). Secondly, *Bifidobacterium* species can ferment sugars that *A. hallii* is unable to ferment (e.g., fucose) and in doing so produce 1,2-propanediol, which can be utilized by *A. hallii* to produce propionate (Schwab et al., 2017). While *A. hallii* is unable to ferment complex oligo- and polysaccharides (Scott et al., 2014), such metabolic compatibilities with *B. adolescentis* likely render *A. hallii* competitive against the 100’s of other species in the gut microbiota.

Overall, similar to case study 1 the SIFR^®^ technology enabled predictive observations as quickly as within days during case studies 2/3. This accurate mirroring of *in vivo* observations that required repeated daily administration over weeks was not restricted to primary substrate degraders (e.g., *B. adolescentis*) but also extended to secondary degraders (e.g., *A. hallii*).

### Technical assessment of the SIFR^®^ technology

#### SIFR^®^ technology has a high technical reproducibility (Technical assessment 1)

At the end of incubation (24 h), the coefficients of variation (CV = standard deviation/average) across the two donor scenarios, each tested in *n* = 6 (one donor and pooled sample of 6 donors) were on average 2.4% for fundamental fermentation parameters (pH, acetate, propionate and butyrate) and 15.2% for quantification of the 50 most abundant microbial species. This covered variation due to reactor preparation, incubation, sampling, but also technical variation of SCFA or sequencing/cell counting analysis. E.g., when butyrate levels in the NSC would be 10.0 mM, the standard deviation would on average be 0.24 mM, meaning that univariate statistical tests with significance level of 0.05 would allow to identify a significant treatment effect when butyrate levels increase up to 10.48 mM (=average + 2 × standard deviation). Similarly, when a microbial species is present at 8.00 log_10_ (cells/mL) in the NSC, a significant effect can be identified when it increases to 8.11 log_10_ (cells/mL). Such high technical reproducibility renders the SIFR^®^ technology highly sensitive in identifying small but significant changes, while enabling studies that focus on biological rather than technical replicates.

#### Accounting for interpersonal differences is essential to provide insights in mode-of-action hypothesis (Technical assessment 1)

**Figure 6:**
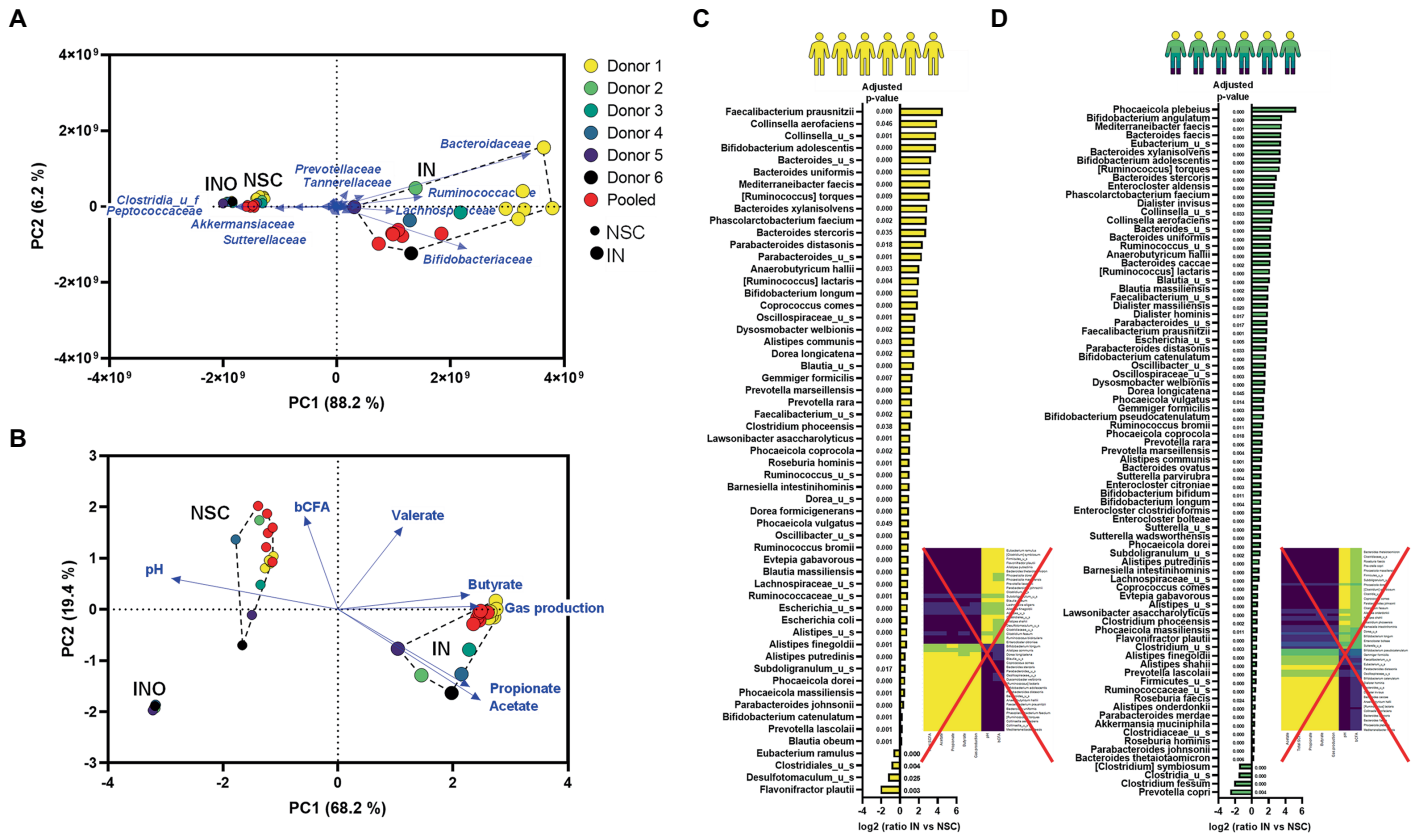
Technical assessment 1/Case study 1: PCA based on microbial composition at family level (cells/mL) **(A)** and fundamental fermentation parameters **(B)** for the *in vivo*-derived inocula (INO) and upon 24 h of incubation in the SIFR^®^ technology (NSC and IN treatment) according to three donor scenarios. IN treatment significantly affected multiple species for both donor strategies [repeated analyses (*n* = 6) from 1 donor **(C)** or from a pooled sample **(D)**] (data expressed as log_2_ (ratio of abundance upon IN treatment compared to NSC), along with the corresponding adjusted *p*-value), correlating with the levels of specific fundamental fermentation parameters (rCCA analysis; threshold >0.8).

PCAs based on microbial composition (Figure 6A) and metabolite production (Figure 6B) revealed that the pooled inoculum did not provide the same results as the average among individual donors. By combining samples of 6 donors, microbial diversity dramatically increased to levels that were not representative for the individual donors (Supplementary Figure S8). This resulted in an overestimation of the metabolic capabilities of the artificial community as reflected by, e.g., total SCFA levels that largely exceeded those of the individual donors (Supplementary Figure S9). Further, a key drawback of pooled samples but also of repetitions from a single donation was that no meaningful correlations could be established between metabolites and species (Figures 6C,D). Using single/pooled donations, all species stimulated by IN correlated with IN-mediated metabolites and vice versa thus impairing to obtain insightful correlations between specific bacteria and metabolites that could support mode of action.

On the other hand, working with a single/pooled sample in *n* = 6 provided great statistical power to identify changes within these samples, which revealed that IN stimulated a broad range of gut microbes. For the pooled sample, 77 (of the 100 most abundant) species significantly increased upon IN treatment (Figure 6D). This number is markedly higher compared to the number of species when accounting for 6 individual samples (Figure 3E). Such general stimulation of a broad range of species also strongly disputes the conclusions of current clinical studies that IN would be highly selective given the small amount of significantly affected taxa [i.e., *Bifidobacteriaceae*; (Vandeputte et al., 2017a)]. The present data shows that counterintuitively, it is not the high but rather the low selectivity of IN that causes only a limited number of taxa (e.g., *Bifidobacteriaceae*) to be significantly affected when accounting for interpersonal differences, especially when only considering proportional and not quantitative sequencing outputs. This finding supports recent hypothesis that IN could not be as selective as has been proposed (Cantu-Jungles and Hamaker, 2020). Overall, the current study highlights the importance of performing *ex vivo* studies in parallel to clinical studies to enable correct conclusions on fundamental mechanistic aspects such as the tentative selectivity of how substrates modulate the gut microbiota, a criterium at the core of the prebiotic consensus definition (Gibson et al., 2017).

#### Mucosal microbiota is enriched with *Lachnospiraceae* in SIFR^®^ (Technical assessment 2)

Upon 24 h of incubation, the mucin gel was preferentially colonized with *Lachnospiraceae*, accounting for, on average, 53.7% of the simulated mucosal microbiota, largely exceeding luminal levels (15.2%; Supplementary Figure S10A). This is in line with human *in vivo* data (Lavelle et al., 2015) and findings from *in vitro* gut models run up to 3 days (Van den Abbeele et al., 2013). In contrast to the current generation of gut models running over weeks that do not preserve enrichment of mucosa-associated *Lachnospiraceae* (Van den Abbeele et al., 2021), the current SIFR^®^ study thus enabled the potential observation of treatment effects on these mucosal microbes. In contrast to the profound treatment effects of IN on the luminal microbiota (Figure 3), IN did not significantly impact mucosal microbes (Supplementary Figure S10B). Treatment effects of IN on the luminal microbiota were similar whether or not the mucin gel was added, both in terms of microbial composition and metabolite production (Supplementary Figures S10C,D). The absence of treatment effects of IN on the mucosal microbiota could potentially be attributed to the aforementioned low selectivity of how IN impacts the microbiota. While it would be relevant to more broadly apply the SIFR^®^ model with mucin gel to gain an understanding of how therapeutics affect this simulated mucosal microbiota, in absence of *in vivo* data on this research topic, it remains difficult to validate the predictivity of such findings.

## Conclusion

The *ex vivo* SIFR^®^ technology generated insights as quickly as within 1–2 days that mirrored the clinical outcomes that required repeated prebiotic intake over weeks, verifying its usefulness in bridging the “Valley of Death” between preclinical and clinical gut microbiome research. Importantly, predictivity was demonstrated for compositional changes down to the species level, not only for primary but also secondary substrate degraders. A fundamental aspect of the SIFR^®^ technology is the accurate preservation of *in vivo*-derived microbiota for the full duration of the experiment, thus classifying the application of SIFR^®^ technology as an *ex vivo* study (rather than an *in vitro* study), which is a study that uses an artificial environment outside the human body with minimum alteration of natural conditions. Another key aspect is the inclusion of multiple donors to account for interpersonal differences. Embracing interpersonal variation is pivotal to establishing correlations that refine the hypothesis on the mode of action for the gut microbiota, while narrowing down the set of significant outcomes to salient features. This increases the relevance of the preclinical findings for the target population, which in turn leads to better decision-making during product development on the transition to clinical studies. By enabling preclinical testing of a larger number of doses, conditions, and combinations in a more predictive set-up, across multiple individuals, and by refining the understanding of the potential mode of action, the success rate of clinical trials could dramatically increase.

The predictive nature of the SIFR^®^ technology opens the door for combined *ex vivo/in vivo* studies where *ex vivo*-obtained insights on endpoints that are intractable *in vivo* (microbiota modulation at site of fermentation, metabolite (e.g., SCFA) and gas production) could enable novel stratification approaches. Another key finding of the study is the ability of quantitative sequencing to remove the bias caused by increased cell density upon prebiotic treatment *ex vivo*, which is crucial to grasp essential aspects such as selectivity of how a prebiotic impacts the microbiota. Astoundingly, the reference prebiotic IN exhibits a very low selectivity in its mode of action, contrary to popular belief among experts (Gibson et al., 2017).

While a key advantage of the SIFR^®^ technology is the absence of a host component, enabling insights that are difficult to obtain *in vivo*, a related drawback is that obtained findings should be regarded as complementary to *in vivo* studies, rather than as potential replacement thereof. In conclusion, *ex vivo* SIFR^®^ technology has the ability to open up patient-tailored microbiome modulation therapies, precluding predictable therapy resistance or adverse health-effects of current treatments. While this *ex vivo* side-step could potentially discharge clinical staff from useless, labor-intensive trails, at the same time, the need for patients to participate in demanding studies would be alleviated, preventing an overall loss of resources.

## Data availability statement

The original contributions presented in the study are included in the article/Supplementary material, further inquiries can be directed to the corresponding author.

## Ethics statement

The studies involving human participants were reviewed and approved by Ethics Committee of the University Hospital Ghent (reference number BC-09977). The patients/participants provided their written informed consent to participate in this study.

## Author contributions

PVdA, CT, CP, DB, EW, MJ, JF, and AB: conceptualization. PVdA: data curation and supervision. PVdA, SD, CT, CP, DB, EW, MJ, JF, DW, and AB: formal analysis and writing—review and editing. PVdA, SD, and DW: investigation. PVdA, SD, CT, CP, DB, EW, MJ, DW, and AB: methodology. CT, CP, DB, and EW: resources. PVdA and AB: project administration. PVdA, SD, MJ, and AB: visualization. PVdA, MJ, and AB: writing—original draft preparation. All authors contributed to the article and approved the submitted version.

## Conflict of interest

PVdA, SD and AB are employees of Cryptobiotix. CT and CP are employees of Roquette, while EW and DB are employees of DSM. DW is employed by CosmosID.

The remaining authors declare that the research was conducted in the absence of any commercial or financial relationships that could be construed as a potential conflict of interest.

## Publisher’s note

All claims expressed in this article are solely those of the authors and do not necessarily represent those of their affiliated organizations, or those of the publisher, the editors and the reviewers. Any product that may be evaluated in this article, or claim that may be made by its manufacturer, is not guaranteed or endorsed by the publisher.
