# Supplementary material for: Bridging preclinical and clinical gut microbiota research using the ex vivo SIFR® technology
Source: Front Microbiol. 2023 Apr 14;14:1131662. doi: 10.3389/fmicb.2023.1131662 (PMC10178071; doi:10.3389/fmicb.2023.1131662)
Supplement: Supplementary file 1 [file Data_Sheet_1.docx]

# Supplementary figures

**Figure** **S1**. (A) Centred PCA based on the (B) levels of the most abundant families (%) in the 18 fecal samples used to inoculate the SIFR^®^ reactors along case study 1 (donation 1-6), case study 2 (donation 7-12) and case study 3 (donation 13-18). Donations 1-6 were also used for technical assessments 1 and 2.

**Figure** **S2**. Proportional sequencing data (%) (A) was converted to absolute data (cells/mL) (B) by multiplying proportions with the total cell count of each sample, as illustrated for samples of case study 2. This results in a large gap between the least abundant species of a given sample (white lines in figure B) and non-detected species (0 cells/mL). A key issue with such gap is that false conclusions on inter-sample comparisons can be made. E.g., when a species represented by the green dot in NSC12 would not be detected in sample RD8, it cannot be concluded whether the treatment (RD) stimulated (green dot “1” in bar RD8) or rather decreased this species (green dot “2” in bar RD8). To avoid false conclusions, a conservative approach was followed during the current project where among the lowest detected values in each sample (indicated by white lines), the highest value (i.e. highest positioned white line) was used as the overall limit of detection (LOD) of the entire dataset (C). All values below this value were replaced by this overall LOD (D). While this approach removes part of the data, particularly for samples with low cell densities (e.g. NSC 7), it ensures that only valid conclusions on treatment effects are made. As a remark, a separate detection limit was established for samples of different origin, i.e. fecal samples (INOx) and SIFR-incubated samples (NSCx and RDx).

**Figure S3. Case study 1:** Fundamental fermentation parameters in the (diluted) *in vivo-*derived inocula (INO) and upon 24h of incubation in the SIFR^®^ technology (NSC and IN treatment). Significant differences between IN and NSC are indicated with */**/*** (0.01 < p < 0.05/0.001 < p < 0.01/ p <0.001).

**Figure S4. Case study 2:** Abundance of those species (cells/mL) that were significantly affected by RD compared to the NSC (FDR = 0.10) in both the *in vivo-*derived inocula (INO) and upon 48h of incubation in the SIFR^®^ technology (NSC and RD treatment) (A). Several species correlated with the levels of specific fundamental fermentation parameters (rCCA analysis; threshold > 0.8) (B).

**Figure S5. Case study 2:** Fundamental fermentation parameters in the (diluted) *in vivo-*derived inocula (INO) and upon 48h of incubation in the SIFR^®^ technology (NSC and RD treatment). Significant differences between RD and NSC are indicated with */**/*** (0.01 < p_adjusted_ < 0.05/0.001 < p_adjusted_ < 0.01/ p_adjusted_ <0.001).

**Figure S6. Case study 3:** Abundance of those species (cells/mL) that were significantly affected by 2’FL compared to the NSC (FDR = 0.10) in both the *in vivo-*derived inocula (INO) and upon 24h of incubation in the SIFR^®^ technology (NSC and 2’FL treatment), along with three additional species that resulted in a marked correlation (*R. gnavus, O. intestinalis* and *B. massiliensis*) (A). Several species correlated with the levels of specific fundamental fermentation parameters (rCCA analysis; threshold > 0.6) (B).

**Figure S7. Case study 3:** Fundamental fermentation parameters in the (diluted) *in vivo-*derived inocula (INO) and upon 24h of incubation in the SIFR^®^ technology (NSC and 2’FL treatment). Significant differences between 2’FL and NSC are indicated with */**/*** (0.01 < p_adjusted_ < 0.05/0.001 < p_adjusted_ < 0.01/ p_adjusted_ <0.001).

**Figure S8. Technical assessment 1/Case study 1:** Cell counts (A) and microbial diversity (Chao 1 (A) and Shannon diversity index (B)) of the original fecal inocula (INO) and upon 24h of incubation in the SIFR^®^ technology (NSC or IN treatment) according to three donor scenarios (one donor (n = 6), 6 donors (n = 1) or a pooled sample (n = 6)). Significant differences between IN and NSC are indicated with */**/*** (0.01 < p < 0.05/0.001 < p < 0.01/ p <0.001).

**Figure S9. Technical assessment 1/Case study 1:** Fundamental fermentation parameters upon 24h of incubation in the SIFR^®^ technology (NSC or IN treatment) according to three donor scenarios (one donor (n = 6), 6 donors (n = 1) or a pooled sample (n = 6)). Significant differences between IN and NSC are indicated with */**/*** (0.01 < p < 0.05/0.001 < p < 0.01/ p <0.001).

**Figure S10. Technical assessment 2/Case study 1:** Several families had a significantly different abundance in the mucin gel compared to the lumen (A). PCA based on microbial composition of the microbiota colonizing the mucin gel (%) upon 24h of incubation in the SIFR^®^ technology (NSC and IN treatment) (B) along with PCAs based on microbial composition of the luminal microbiota at family level (cells/mL) (C) and fundamental fermentation parameters (D), both in presence (M; technical assessment 1) and absence (case study 1) of the mucin gel.

# supplementary tables

**Table** **S1**. **Donor information and BSS for all samples**. Gender and age of the donors that provided the 18 donations (with a specific BSS) used for SIFR^®^ incubations along case studies 1, 2 and 3.

| **Donation** | **Case study** | **Gender** | **Age (y)** | **BSS** |
| --- | --- | --- | --- | --- |
| 1 | 1 | F | 28 | 3-4 |
| 2 | 1 | M | 30 | 5 |
| 3 | 1 | M | 25 | 3-4 |
| 4 | 1 | M | 37 | 3 |
| 5 | 1 | M | 29 | 4 |
| 6 | 1 | M | 37 | 4 |
| 7 | 2 | F | 39 | 3 |
| 8 | 2 | F | 28 | 4 |
| 9 | 2 | F | 49 | 5 |
| 10 | 2 | M | 25 | 4 |
| 11 | 2 | M | 60 | 4 |
| 12 | 2 | M | 50 | 3 |
| 13 | 3 | M | 28 | 3 |
| 14 | 3 | F | 30 | 4 |
| 15 | 3 | F | 28 | 4 |
| 16 | 3 | M | 37 | 3 |
| 17 | 3 | M | 29 | 3 |
| 18 | 3 | M | 29 | 3 |
